# Investigation of Low Heat Accumulation Asphalt Mixture and Its Impact on Urban Heat Environment

**DOI:** 10.1371/journal.pone.0133829

**Published:** 2015-07-29

**Authors:** Jianguang Xie, Zhaoxu Yang, Leilei Liang

**Affiliations:** Department of Civil Engineering, Nanjing University of Aeronautics and Astronautics, Nanjing, China; Università di Trento, ITALY

## Abstract

This study is focused on investigating the effectiveness of low heat accumulation asphalt mixture and its impact on the urban heat environment. Infrared radiation experiments showed that the temperature of the asphalt mixture decreased with the increase in far-infrared radiant material. The results also revealed that, compared to asphalt with 0% far-infrared radiant content, the asphalt material with a certain ratio of far-infrared radiation material had higher stability at high and low temperatures as well as good water absorption capacity. The Marshall stability of the specimen mixed with 6% far-infrared radiant was higher by 12.2% and had a residual stability of up to 98.9%. Moreover, the low-temperature splitting tensile strength of the asphalt mixture with 6% far-infrared radiation material increased by 21.3%. The friction coefficient of the asphalt mixtures with 6% and 12% far-infrared radiation material increased by 17.7% and 26.9%, respectively.

## Introduction

The urban heat island (UHI) effect is the phenomenon in which the temperature of an urban area is significantly higher than those of the suburban areas. The temperature variations in the suburban areas are also far lower than the changes in urban areas [1, 2]. The average temperature of a UHI in urban area is usually higher than the annual average temperature of suburban areas by more than 1°C. It has been reported that cities in China, e.g., Nanjing, Wuhan, and Chongqing, are the hottest cities, with summer temperatures often increasing to over 40°C, and that the urban heat island effect plays a key role in this temperature rise [3]. This has become the main factor restricting the sustainable development of the socio-economic environment in these cities [4].

Many researchers have found that the heat-absorption capacity of roads and other building materials is higher than that of green belts and water bodies and that the surface temperature around asphalt pavements increases in summer mainly because of their low heat-reflection capacity and high heat-absorption rate [5, 6]. The urban heat island effect can be reduced by using effective reflective materials in the construction of buildings, pavements, and roads. Asphalt has been considered as a good material for heat reflection when mixed with more-reflective infrared materials [7].

The temperature sensitivity of asphalt is stronger under extreme temperature conditions, and its strength, stability, and performance are adversely affected by high temperatures, leading to cracks in the pavement or road surface and damage due to surface water and seepage. Therefore, there is a need for improving asphalt mixtures to meet the requirements of stability at high and low temperatures, water stability, and traffic-load resistance, which are important for their practical application. To this end, low heat accumulation asphalt mixture was investigated in the present study. Low heat accumulation asphalt is a new kind of pavement material, prepared by mixing asphalt with infrared powder. Previous studies have showed that crushed stone mastic asphalt (SMA) is a high-grade road pavement material because of its excellent skid resistance and rutting resistance [8–10]. SMA is a mixture of asphalt, fiber stabilizer, and a small amount of fine aggregate consisting of mastic asphalt binder that fills in the gaps of the coarse aggregate graded skeleton [11–14]. SMA has a strong permanent deformation resistance and is capable of resisting deformation at high temperatures, which also significantly improves the water stability of the mixture.

Heat energy over pavements is transformed into radiant heat energy, and part of this energy radiates into the atmosphere within the infrared wavelength band if the asphalt material is mixed with a certain amount of infrared powder. Because of the low absorption rate of infrared wavelengths by atmosphere, the radiant heat energy released over the earth surface above roads incorporated with infrared powder would be significantly reduced. The variations in temperature distribution measured by various test methods can provide the basic knowledge required for the application of the asphalt mixture. A wide range of mix design methods are available worldwide, but the Marshall method, which is in accordance with national standards, is most commonly used in China because of the domestic conditions, experience, and technical aspects [8, 15]. This method has been improved with the advancements in the international design experience. However, it also requires the use of other methods for the Marshall test, according to the mix design specifications in JTG F40-2004 *Technical Specifications for Construction of Highway Asphalt Pavements* [16].

The current study aims at investigating the modified asphalt mixture to analyze its stability, performance, and suitability for reflecting solar radiations. Ordinary asphalt material and modified asphalt material, far infrared radiant material (FIRM) were examined to study their cooling properties, strength, and stability, in order to ensure the requirements of their practical application.

## Experimental Procedure

### Test Material

Low heat accumulation asphalt mixture is mixed with ordinary asphalt mixture to replace a part of the limestone slag powder with infrared powder as filler. Because of the physical differences between the infrared powder and the slag, the replacement inevitably leads to changes in the best Whetstone size ratio. Therefore, different SMA and infrared powder mixture ratios were investigated, based on the optimum asphalt content obtained from the Marshall method, which provides a guideline for the production and engineering applications of asphalt specimens. For the selection of far-infrared radiant mixture, the infrared emissivity of infrared powder was measured. Then select a wave band from 8–14 μm band width which showed higher emissivity for infrared powder. This powder was then mixed with asphalt to prepare the far-infrared radiant mixture. This mixture showed very effective cooling effect. All experiments in the current study were carried out in replicates to ensure the accuracy. The climatic conditions in Jiangsu Province of China are humid and hot, so low-grade asphalt should be selected for an appropriate balance between the high and low temperature requirements. Therefore, the asphalt material used in the current study was SK-A grade 70# from South Korea. The technical specifications of the asphalt material are listed in Table 1.

**Table 1:** Technical specifications of asphalt.

Technical indexes	Penetration degree (at 25°C, 5 s, 100 g)	Softening temperature	Ductility (at 15°C)	Density (at 25°C)
**Unit**	0.1mm	°C	cm	g/cm^3^
**Test results**	67.8	52.4	120	1.034
**Code requirements**	60∼80	<46	<100	>1.01

### Packing Materials

#### Slag and limestone ore

Slag and limestone aggregate were added to the mixture to increase the density and adhesion, respectively, of the asphalt.

#### Synthetic infrared modified powder

Domestic synthetic modified ceramic powder (known as Lianjia powder or LJ) was used in this study because of its low cost, compared to other widely used powders. LJ infrared ceramic powder is a synthetic black-colored powder with a mesh size of about 700~800 openings per square inch and a high infrared-emission rate. A modified alkali treatment using Ca(OH)_2_, as the base reagent, and water in a 1:2 ratio was carried out to increase the alkalinity of the LJ infrared powder, so that the adhesion of the asphalt material could be improved. The infrared powder was soaked in the base solution for about 3 h and then transferred to an oven to dry to get a constant weight for the tests.

#### Mineral aggregate and mix design

Basalt gravel aggregates, which are mostly black, hard, and have a porphyritic structure with 7 to 7.5 Mhos hardness, were chosen in the current study as they play a very important role in the skeleton. Lignin fibers were also used in current study. As the temperatures in the chemical processing of lignin fibers go up to 250°C, they can be very stable, basic, and solvent and have been used to improve skid resistance in highway pavements. The key properties of lignin fibers include reinforcement effect, dispersion, adsorption, and absorption, stabilizing effect, and bonding effect. Mix design specimens were prepared using SMA-13 as the base material and modified infrared powder as the filler, equivalent to the slag and limestone ore. The physical parameters of the selected lignocellulosic fiber are listed in Table 2.

**Table 2:** Physical parameters of lignocellulosic fiber.

**Length**	<6mm	**Ash content**	18 ± 5%
**pH**	7.0 ± 1.0	**Oil absorption rate**	no less than 5 times the fiber’s weight
**Water content**	<5%	**Heat tolerance**	230°C (reach 280°C in short time)

**Fig 1:**
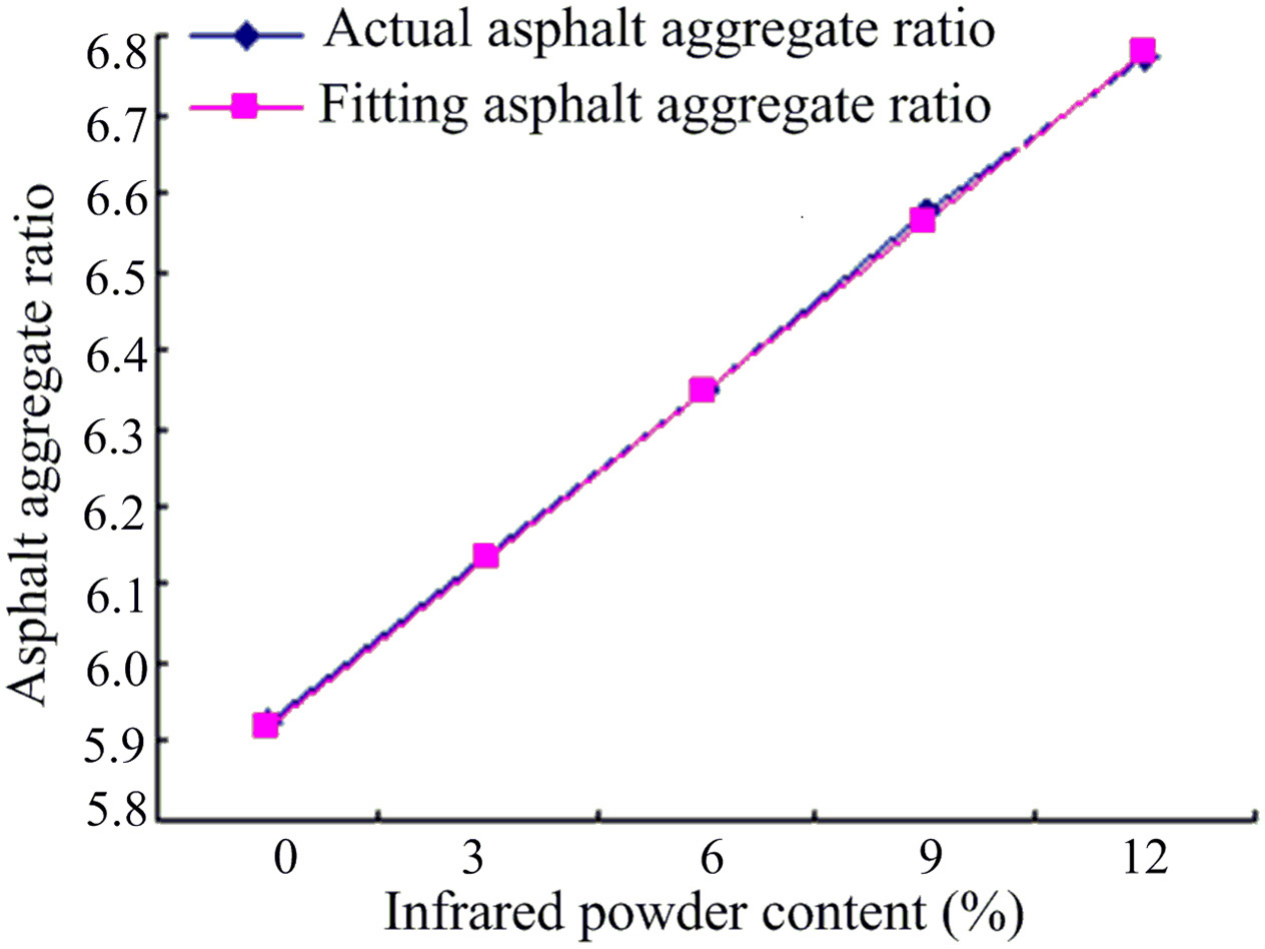
Relationship between optimum asphalt aggregate ratio and infrared powder content.

The equipment used in the study included an automatic screening instrument, controllable oven, electric compactor, stability tester, rutting tester, and incubator. The physical properties were analyzed by various tests such as coarse aggregate test, infrared emissions rate test, and cooling performance test. Mix design experiments were carried out, in accordance with JTJ 052–2000 *Standard Test Methods of Bitumen and Bituminous Mixtures for Highway Engineering* [17] and the Marshall test, to determine the aggregate gradation, fiber content, density, and optimal ratio of whetstone. Fig 1 shows the actual asphalt aggregate ratios, from the whetstone test, and the fitted asphalt aggregate ratios for different infrared powder contents. The optimum asphalt mixture ratio shows a linearly increasing trend with the infrared powder content; hence, a straight line could be fitted to the curve of optimum asphalt ratio (y) and infrared powder content (x), represented by Eq 1. The calculated correlation coefficient was 0.9998, which shows the effectiveness of the infrared mix material.

$$Y=5.926+\frac{21.35X}{3}$$ (1)

where Y is the best whetstone ratio, and X is the infrared powder content (0–12%).

### Infrared Radiation Test (Cooling Performance Test)

An infrared lamp (IR lamp) heating device was used for the insulation process of the Marshall specimens, which had an insulation tank with a groove depth of about 63.5 mm and diameter of about 101.5 mm. A hole was created at the center of the insulation tank bottom to insert the infrared thermometer probe. All the specimens were placed in a constant-temperature bath to reach an initial temperature of 25°C. An infrared temperature gun was used to measure the surface temperature, while a numerical thermometer was used to measure the temperature of the bottom surface of the test material.

The Marshall specimens were prepared using the optimum asphalt ratios, calculated by Eq 1, for infrared powder contents of 0%, 3%, 6%, 9%, and 12%. The test material was cooled at room temperature, but because it is hard to avoid the contamination of specimen surface, they were encapsulated in plastic wrap and placed in a water bath at 25°C for isothermal curing. Dense packaging was used to prevent water vapor from entering the interior of the specimens. After 24 hours, the specimens were taken out from the water bath tanks, stripped of their dense packaging, and placed on a metal frame holding the stage to be tested with an infrared temperature probe.

The IR lamp was kept at a vertical distance *H* from the surface of the specimen, which was adjusted to 20 cm. The asphalt material was kept under the infrared light for 61 minutes to reach the equilibrium temperature. Taking into consideration that urban road temperature can rise up to 68°C in extreme summers, the equilibrium temperature was calibrated to be 68°C by adjusting *H* using the differential method. Infrared temperature gun was fixed on a stand so that the infrared indicated the center of the specimen surface. Initial temperatures of the upper and lower surfaces of the specimen were simultaneously measured at this center position.

## Results and Discussion

### Surface Heating and Cooling Experiment

**Fig 2:**
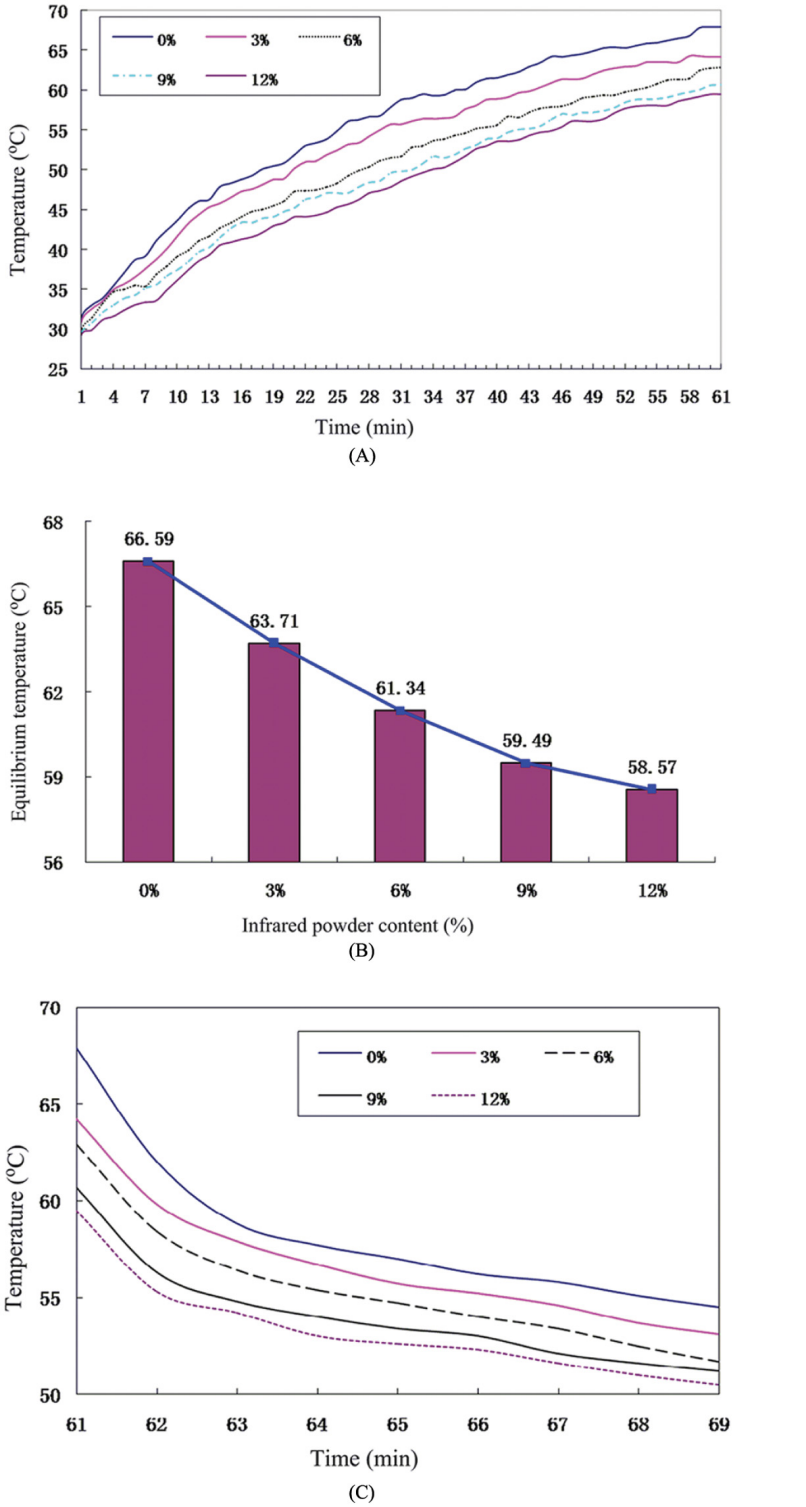
Surface heating and cooling performance of asphalt mixture under different infrared powder content. A. Surface heating temperature; B. Surface equilibrium temperature; and C. Surface cooling temperature.

The experimental results showed that, with the increase in irradiation time, the surface temperature of the specimen gradually increased and became more balanced with time (Fig 2A). To ensure accuracy, the specimens were analyzed for 52~61 minutes when the average temperature on the surface of the specimen was at equilibrium temperature. It was found that the equilibrium temperature of the specimen decreases with the increase in infrared powder content (Fig 2B). At 0% infrared powder content, the specimen temperature reached 66.59°C, which is close to the actual temperature of roads during extreme summers. The equilibrium temperature was noted to be 63.71°C, 61.34°C, 59.49°C, and 58.57°C for infrared powder contents of 3%, 6%, 9%, and 12%, respectively. Compare between infrared powder contents of 0% and 12%, the difference of equilibrium temperature was 8.02°C. It is clear from the findings that the far-infrared powder can be incorporated into the asphalt mixture to reduce the temperature of the specimen surface and improve the high-temperature stability of the mixture.

Fig 2C shows that the surface of the specimens in contact with air has relatively low temperatures in the absence of the infrared radiation. The results show that the temperature drops with time for all the FIRM specimens and that the surface of the specimens with higher infrared powder contents decreased to lower temperatures. This is because the specimens incorporated with the far-infrared powder absorbed less energy when irradiated with infrared light, and hence had lower heat accumulation.

### Bottom Heat Experiment

**Fig 3:**
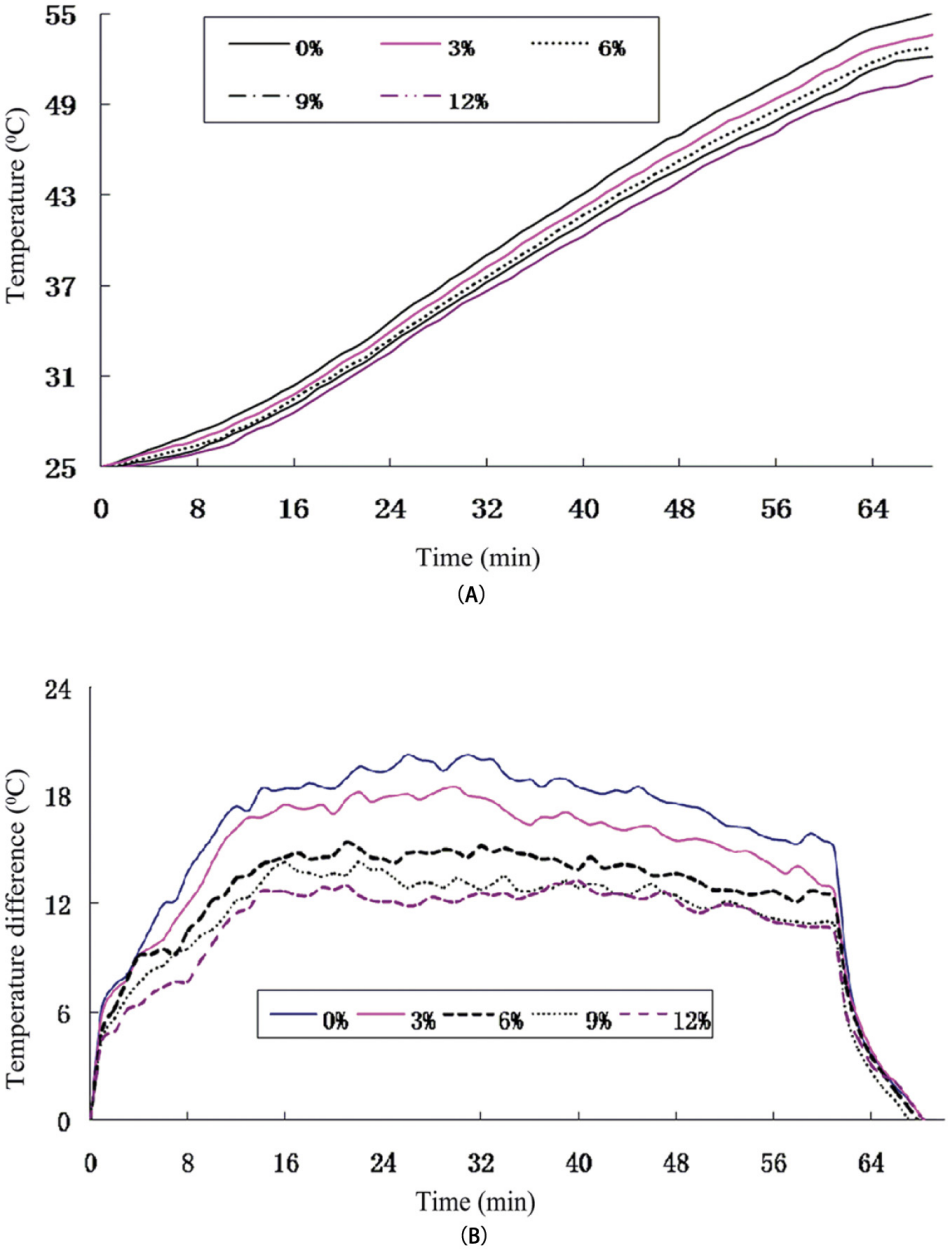
Bottom heating performance of asphalt mixture under different infrared powder content. A. Bottom heating temperatures; and B. Residual temperature difference.

The bottom of the material was still hot after the infrared radiation was turned off because more heat was absorbed by the specimen’s bottom surface relative to the insulated top surface. It is clear from Fig 3A that the rise in bottom temperature was reduced with the increase in infrared powder dosage. Fig 3B shows the temperature difference between the upper and lower surfaces, and it can be seen that the temperature difference was lower for specimens with higher infrared powder contents. This trend is in accordance with the principles of heat transfer, namely change in the temperature gradient and differences in the thermal conductivity.

### High-Temperature Stability

Asphalt is a typical rheological material, and its strength and stiffness modulus decrease with increasing temperatures. Therefore, the stability of the asphalt is not guaranteed if the temperature rises to very high levels. Many factors, such as high temperatures and heavy traffic loads, can affect the stability and performance characteristics of the asphalt pavement including reflection of pavement heat, rutting, folding, and pavement life. Therefore, the high-temperature stability of asphalt pavements needs to be improved, especially for use in areas that have hot summers.

The test methods currently used in China and internationally to study the temperature deformation characteristics of asphalt can be roughly divided into three categories: empirical test methods such as the Marshall test, relevant performance test methods such as the indoor rutting test, and mechanics-based test methods such as uniaxial creep and uniaxial compression tests [18]. Of these methods, the Marshall stability test and rutting test are the most commonly used ones. In order to investigate the high-temperature stability properties of the low heat accumulation asphalt mixture, the Marshall stability test was carried out, and the Marshall stability, flow, and Marshall modulus values were measured.

### Marshall Stability

**Fig 4:**
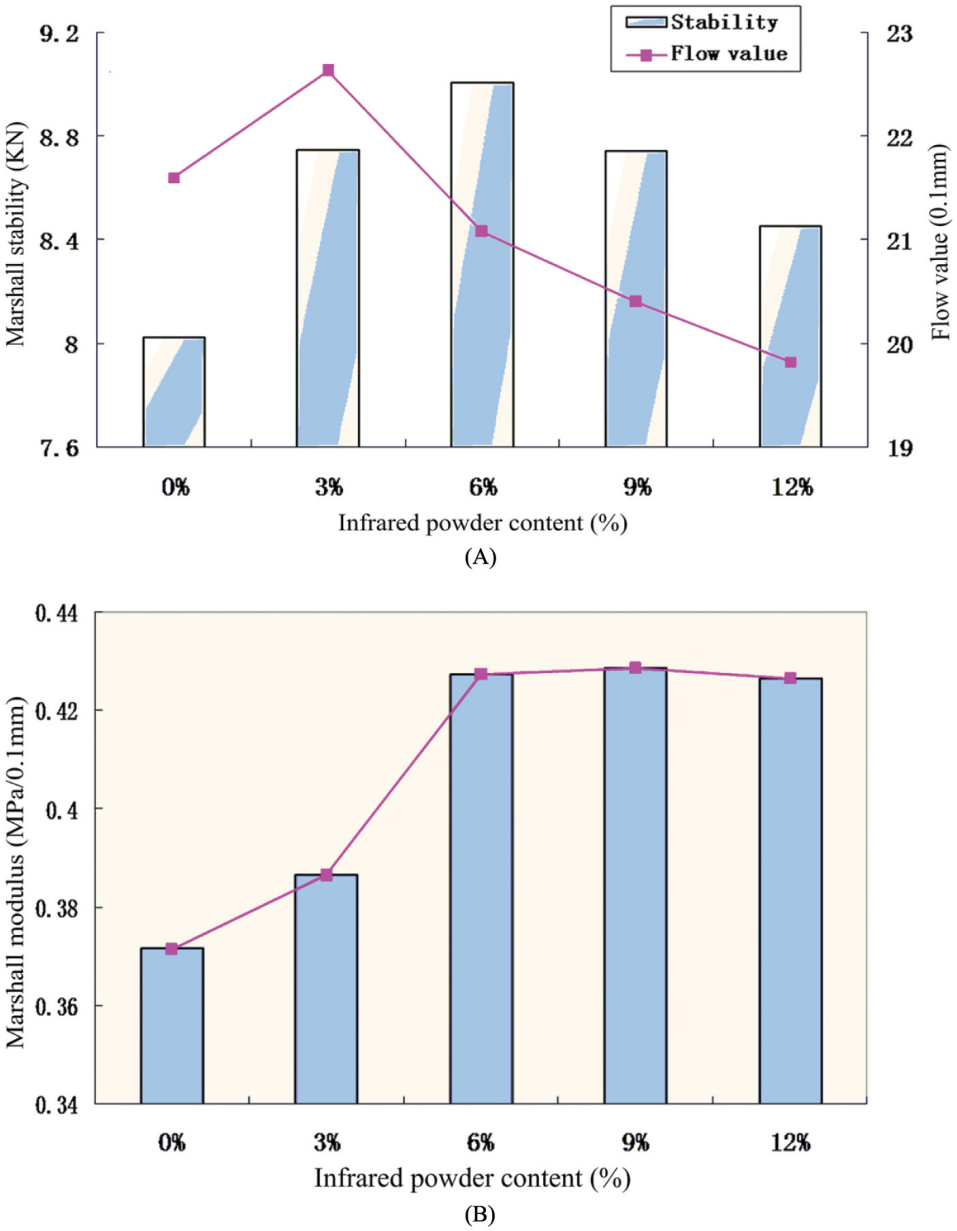
Marshall stability test of asphalt mixture. A. Asphalt mixture Marshall stability test; and B. Relationship between marshall modulus and infrared powder content.

Results from the current study revealed that the flow values for the five asphalt mixtures (Fig 4A) were in accordance with the regulatory requirements of 15~40 (0.1 mm) from JTG F40-2004 *Technical Specification for Construction of Highway Asphalt Pavements* [16]. According to these specifications, the Marshall stability must be greater than 8 kN for asphalt highway traffic in hot summers. The results show that the Marshal stability was higher for the mix with 3% infrared powder, but it decreased with increasing infrared content in the range from 5.3% to 12.2%. Overall, the Marshal stability was found to be low in all the mixing ranges. Fig 4B shows the relation between the Marshall modulus and infrared powder content. The Marshall stability and flow values reflect the strength and deformation properties of the mixture, and the Marshall modulus is the ratio of the two. The results show that the average Marshall modulus improved to some extent when the infrared powder content was more than 6%, reaching a value of 0.427 MPa/0.1 mm. Therefore, the incorporation of far-infrared powder can improve the strength of the asphalt mixture as well as its high-temperature stability.

### Low-Temperature Stability

At low temperatures, low shrinkage cracks are easily produced in asphalt pavements, affecting the normal use of the road as well as its durability. Split test, contraction coefficient test, and stress relaxation tests are the commonly used test methods to investigate low-temperature stability [19]. These test methods mainly rely on two mechanical modes, namely deformation at low temperatures and exposure to low-temperature fracture tension. To examine the low-temperature bottom crack resistance of the low heat accumulation asphalt mixtures, splitting tensile strength cryogenic tests were carried out in accordance with JTJ 052–2000 *Standard Test Methods of Bitumen and Bituminous Mixtures for Highway Engineering* [17]. The test mixture had to withstand a temperature of around -10°C at a loading rate of 1 mm/min. Under normal circumstances, the temperature during the winter season is around 0°C. Taking this into account, -10°C was selected as the test temperature to analyze the low-temperature crack resistance of the specimen under cryogenic fracturing, using a salt-solution immersion method.

**Fig 5:**
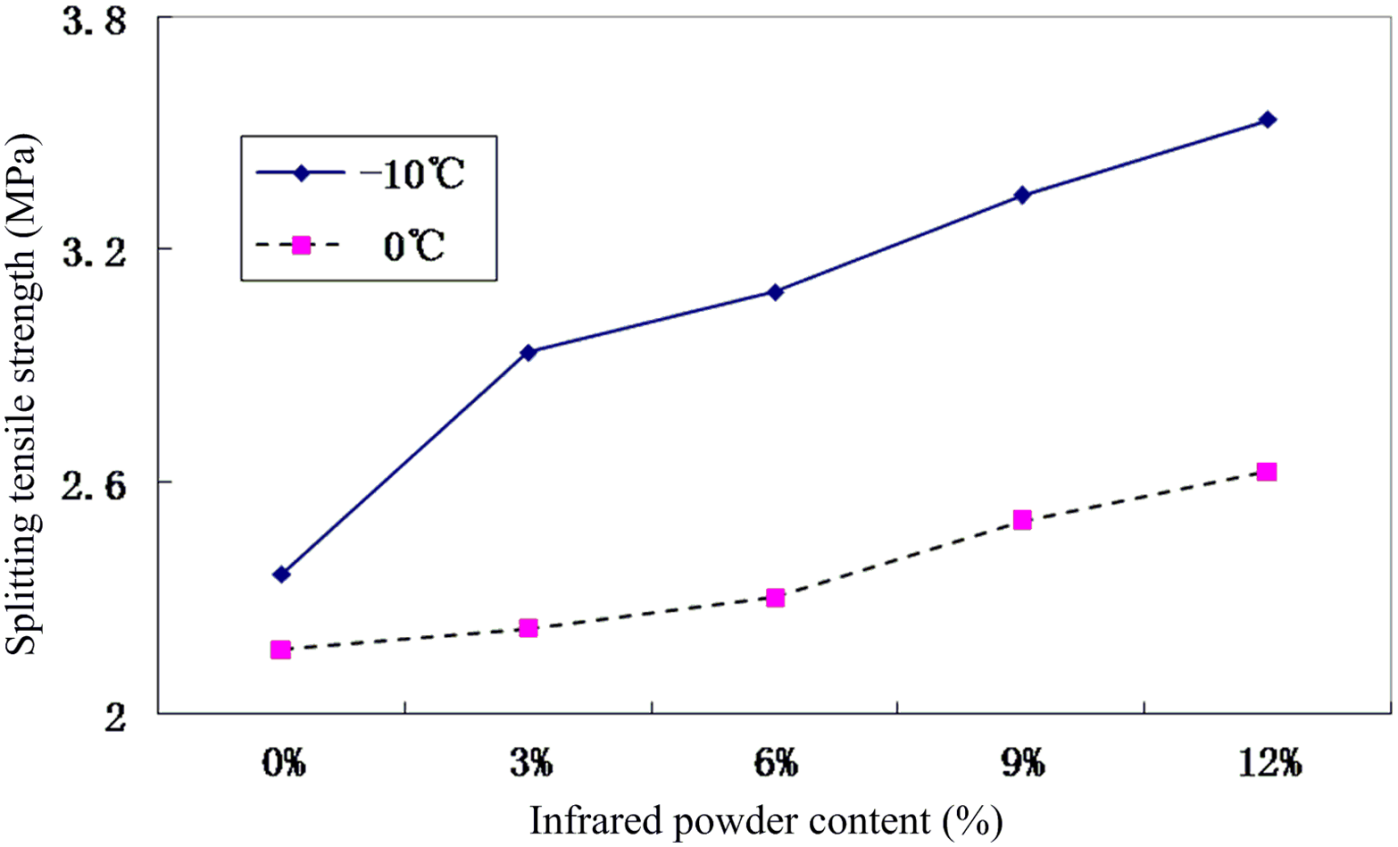
Relationship between splitting strength and infrared powder content.

The salt concentration of the solution was determined at -10°C, using difference approximation for the freezing point, and was to be 12.6%. Fig 5 shows that the splitting strength gradually increased with the increase in infrared powder content at both 0°C and -10°C temperature conditions. The 0°C low-temperature stability tests show that the splitting tensile strength was increased by 2.6%, 6.2%, and 15.33% when the infrared powder content was 3%, 6%, and 9%, respectively, compared to the value at 0% infrared powder content. When the infrared powder dosage increased to 12%, the highest splitting tensile strength of 2.626 MPa was obtained, an improvement of 21.3%. Hence, it is clear from these results that the incorporation of infrared powder can improve crack resistance of the asphalt mixture at 0°C low-temperature conditions. The results of the splitting test at -10°C show that the splitting tensile strength at infrared powder contents of 3%, 6%, 9%, and 12%, was increased by 24.2%, 30.8%, 41.42%, and 50.1%, respectively, an enhancement of 2.6%, 6.2%, 15.33%, and 21.3%, compared to the values at 0°C. It is clear from these results that the infrared powder improves the performance of the asphalt mixture significantly at low temperatures. From the test results (Fig 5), it can be concluded that the splitting tensile strength of ordinary asphalt material can be improved under both low-temperature conditions (0°C and 10°C) by mixing 7% of infrared powder, thereby increasing the crack resistance of the asphalt pavement.

### Water Stability

The water stability evaluation method for asphalt mixtures was developed based on the analysis of the mechanism of water damage. The Marshall specimen immersion residual stability test was used in this study to evaluate the water stability performance of the asphalt mixture at low-temperature conditions. Previous studies have found that the water stability performance of asphalt mixtures is affected by several factors such as the nature of the raw materials, apparent pH, fiber content, and admixtures [20, 21]. The test results show that the mix asphalt content has a significant impact on the water stability and that under normal circumstances, the water content increases the stability and performance of the mix asphalt mixture when Whetstone ratio reaches to a certain level. The water stability was enhanced by the incorporation of an appropriate amount of wood fibers, and the performance of the water-stable mixture significantly improved after the addition of alkaline additives, which increase the adhesion between the asphalt and aggregate materials. The results from the residual stability test showed that 0.4% wood fiber, limestone, and slag powder improves the water stability of the mixture after alkali treatment with infrared radiations. It was also found from the results that the addition of 6% infrared powder to the asphalt mixture increased its residual stability to more than 99%.

Analysis showed that increasing the dosage of infrared powder and alkali treatment increase the reaction between the surfactant mixture and the asphalt material, significantly improving the bond strength between the mineral aggregate and asphalt. When the infrared powder content of the mix was 6%, the residual stability remained at around 99%. This test not only showed the water stability trend as a whole, but also the ratio of powder binder and basic ingredients required to reach the peak performance. Hence, the low heat accumulation asphalt mixture has a higher residual stability and greater water stability to meet the performance requirements for use in road construction.

### Texture Depth and Friction Coefficient

**Fig 6:**
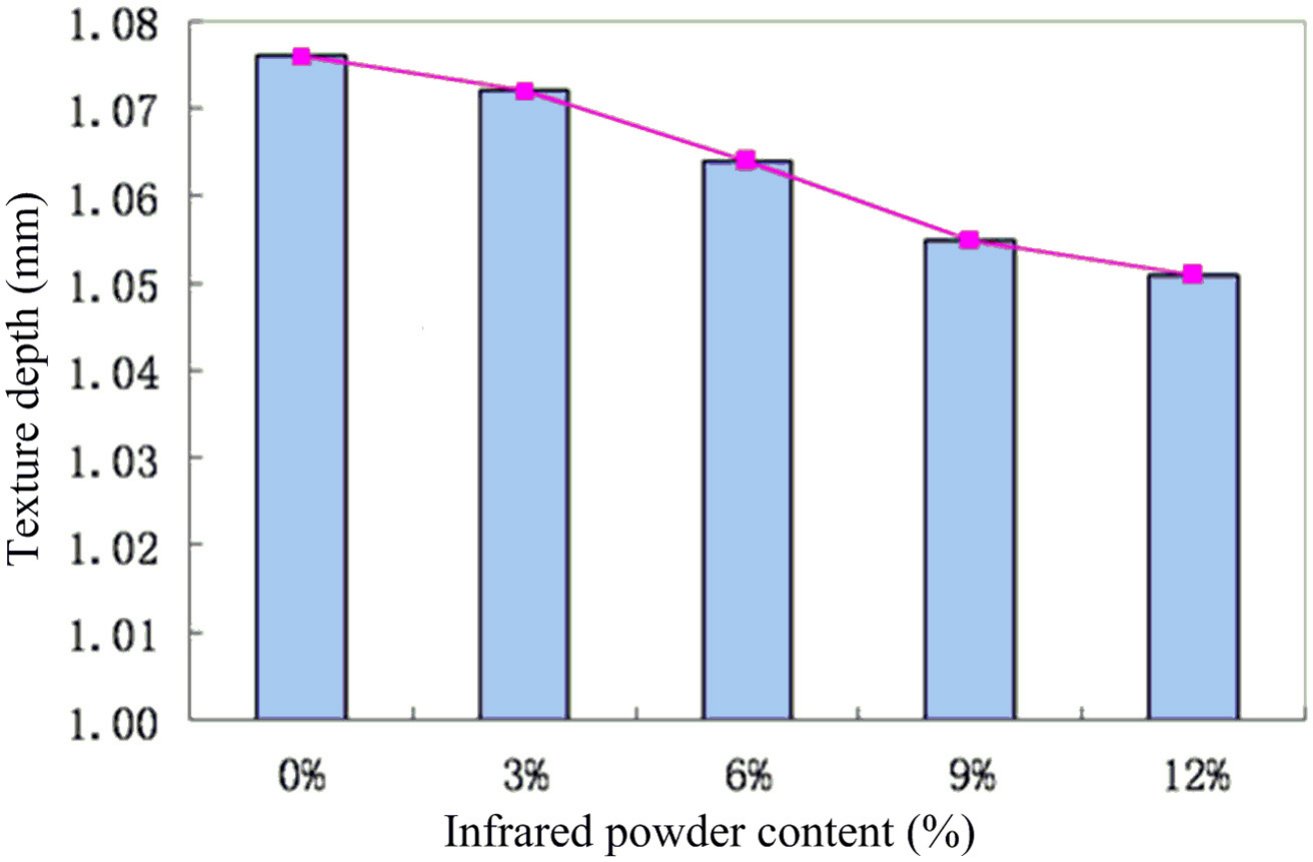
Relationship between texture depth and infrared powder content.

Pavement texture depth and skid resistance play a significant role in high-speed driving and performance of the road pavement [22]. The relationship between texture depth and infrared powder content is shown in Fig 6. It can be seen that the depth of the surface structure of asphalt mixture for the five specimens reached 1.05 mm, which is much greater than the texture depth of ordinary AC AK asphalt pavement. Under normal circumstances, the depth of the asphalt pavement structure is about 0.7 mm. Analysis of the dense structure of the SMA skeleton compared to the AC suspension structure could be very helpful in improving skid resistance.

The surface structure depth of the five asphalt mixtures was tested in order to meet the SMA pavement standard specifications, which require the texture depth to be higher than 0.8 mm and lower than 1.3 mm. The findings showed that the mix grading design meets the required standard of 1 mm and has reasonable skid resistance to meet the engineering requirements. The results also showed that the surface texture depth decreased slightly with the increase in infrared powder content. The surface structure depth was 1.08 mm when the infrared powder content was 0% and 1.05 mm when the infrared powder content reached 12%, decreasing by 2.32%. This trend is associated with the increased porosity of the mixture and decrease in the aggregate ratio due to increased filling of gaps in the structural asphalt road surface. The porosity of the mixture decreased from 3.4% to 2.9% with the increase in infrared powder content, causing a slight decline in the depth of the pavement structure.

**Fig 7:**
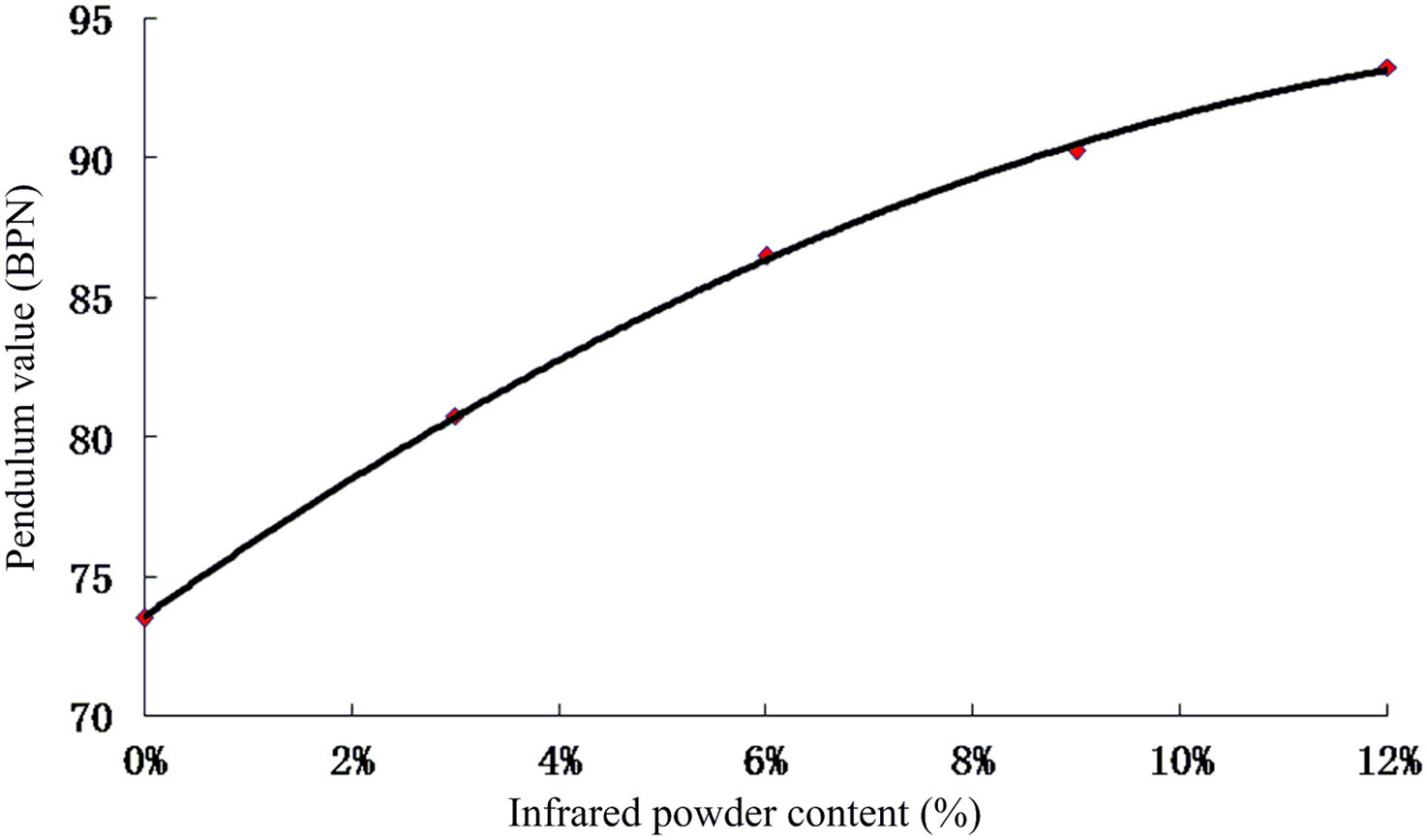
Relationship between pendulum value and infrared powder content.

The rut-simulation test was used to determine the longitudinal coefficient of friction of the test specimens. The test was carried out disregarding the impact of the external environment, size of the main road, road surface friction coefficient of roughness, and frictional properties of materials related to the asphalt. At the macro level, when the surface roughness was higher, the surface texture and friction coefficient were also high. At the micro level, a higher mix of textures in the aggregate resulted in a stronger asphalt mastic adhesion and higher friction coefficient. Fig 7 shows the fitted relationship between the longitudinal friction coefficient and the infrared powder content. It can be seen that the selected graded asphalt friction coefficient reached 0.735 (% pendulum value), which is higher than the road index value of 0.54. The friction coefficient was enhanced by the addition of infrared powder to the asphalt, increasing by 17.7% and 26.9% at 6% and 12% infrared powder contents, respectively, compared to the value at 0% infrared powder content. The reasons for the slight decrease in the depth of the surface structure and increase in the friction coefficient could be the following: 1. The asphalt mastic viscosity increases after the alkali treatment of the infrared powder, which increases the friction; 2. The strong performance of the infrared absorption powder dispersed in the asphalt ensures that the inside surface of the mixture does not overflow, which is beneficial to its anti-slide lasting stability properties. It was also found in a previous study that the alkaline treatment improves the adhesion of the asphalt material and that mineral aggregate in the asphalt surface produces chemical components, which rearrange to form the structure of the asphalt, increasing the viscosity and the friction coefficient [22]. Hence, the performance and long-term stability of the pavement are improved.

## Conclusions

This study was aimed at investigating the effectiveness of low heat accumulation asphalt mixture and its impact on the urban heat environment. The infrared radiation asphalt mixture prepared in different proportions has low heat storage capacity. In this paper, the cooling performance of the infrared radiation asphalt mixture was also studied. The following conclusions were made:

1. The mixed proportion design experiment showed that there is a linear correlation between the optimal asphalt content and the far-infrared radiation material content (Eq 1). The optimal asphalt content was increased by 0.2135% with the increase in far-infrared radiation material content by 3%.
2. Infrared radiation experiments showed that the asphalt temperature decreased with the increase in far-infrared radiation material content. The results also revealed that, compared to the asphalt with 0% far-infrared radiation material content, the asphalt material with a certain ratio of far-infrared radiation material has higher stability at high and low temperatures as well as good water absorption capacity.
3. The Marshall stability of the asphalt mixture increased by 12.2% with the addition of 6% far-infrared radiation material, and its residual stability increased to 98.9%.
4. The low-temperature splitting tensile strength of the asphalt mixture increased by 21.3% with the addition of 6% far-infrared radiation material
5. The friction coefficient of the asphalt mixtures with 6% and 12% far-infrared radiation material increased by 17.7% and 26.9%, respectively, compared to the mixture with no far-infrared radiation material.
